# Suspected Neratinib Macular Toxicity Presenting As Macular Telangiectasia Type II

**DOI:** 10.7759/cureus.33964

**Published:** 2023-01-19

**Authors:** Moises Enghelberg, Syeda Kaifee

**Affiliations:** 1 Retina Service, Loma Linda University Eye Institute, Loma Linda, USA; 2 Ophthalmology, Lake Erie College of Osteopathic Medicine, Elmira, USA; 3 Ophthalmology, Rochester Regional Health, Rochester, USA

**Keywords:** retina, chemotherapy-related toxicity, retinal toxicity, maculopathy, tyrosine kinase receptor inhibitors

## Abstract

The purpose of this case report is to present the first case of neratinib maculopathy. We describe the initial presentation, baseline characteristics, imaging findings, and outcomes. The case report is accompanied by a thorough literature review including possible mechanisms of tyrosine kinase inhibitor (TKI) maculopathy.

Neratinib is a novel TKI that is commonly used in the treatment of breast-associated malignancies. Neratinib toxicity presents similarly to macular telangiectasia type II but differs with the fine granular hypofluorescent areas spanning the limit of the posterior pole and vascular arcades as well as the nasal aspect of the optic nerve.

We report a case of suspected macular toxicity secondary to neratinib. Concomitant use of neratinib in conjunction with docetaxel and other chemotherapeutics with known retinal side effects should alert clinicians of an increase in the risk of macular toxicity. Albeit commonly reported ocular side effects of TKIs, maculopathy is a rare and potentially overlooked side effect. Patients that have planned chemotherapy should undergo a baseline retinal examination.

## Introduction

The ability of anticancer therapies to cause ocular toxicity might be widely under-appreciated. In recent years, the advent of myriad novel chemotherapeutic agents of different classes has given retina specialists the task of recognizing and describing the retinal side effects of these medications. Ocular toxicity has been a well-established side effect of mitogen-activated protein kinase (MEK) inhibitors and taxane therapy. Tyrosine kinase inhibitors (TKIs) are not often associated with macular toxicity and are not usually employed in the treatment of breast malignancies [1-3]. This case report discusses the increased risk of macular toxicity arising from TKI therapy, as well as the maculopathy that occurs with concomitant TKI and taxane therapy. 

Tyrosine kinase inhibitors target an enzyme responsible for downstream signal transduction of cell growth, division, differentiation, apoptosis, and death. Neratinib is a specific TKI that phosphorylates epidermal growth factor (ErBb) receptor family 1, ErBb2, and ErBb4 and inhibits signal transduction via covalent bonding on cysteine residues on adenosine triphosphate (ATP)-binding domains of HER1, HER2, and HER3 in breast cancer. The retinal pigment epithelium expresses ERBB1 and ERBB2 receptors which could be a possible site for toxicity for this drug class. Interestingly, the role of tyrosine kinase signaling has been detailed in the avian retina. In animal models, the presence of retinal-expressed kinase (Rek) during development in the avian population affects the development of Müller cells and glial cells [4].

The most commonly described ocular side effect of TKIs is disruption of the ocular surface [5]. We describe the features of macular toxicity seen with neratinib therapy. In this case, macular toxicity due to neratinib use presents findings reminiscent of macular telangiectasia type II (MacTel type II) making Müller cell toxicity of mechanistic interest.

## Case presentation

The patient is a 61-year-old Hispanic woman with a history of left breast cancer, diabetes mellitus type 2, and hypertension. The patient's medication history before her diagnosis of breast cancer included metformin 500mg twice a day (BID) orally (PO), and Lisinopril 10mg PO daily. She initially presented with left breast pain in 2018 and went to the emergency department. A mammogram was performed revealing a 1.2 cm hypoechoic cystic lesion in the left breast in the periareolar region. A biopsy was performed and determined to be a malignant invasive ductal carcinoma grade 3 out of 3 estrogen receptor (ER)/progesterone receptor (PR) negative HER-2 positive. The patient underwent a left modified radical mastectomy with sentinel lymph node dissection. Pathology showed a 1.8 cm grade 3 invasive carcinoma, pT1c, N0, M0, ER negative, PR negative, and HER2 positive. The patient underwent six cycles of docetaxel, carboplatin, and trastuzumab. She also received a year-long course of trastuzumab. Afterward, the patient started on neratinib 200mg daily.

**Figure 1:**
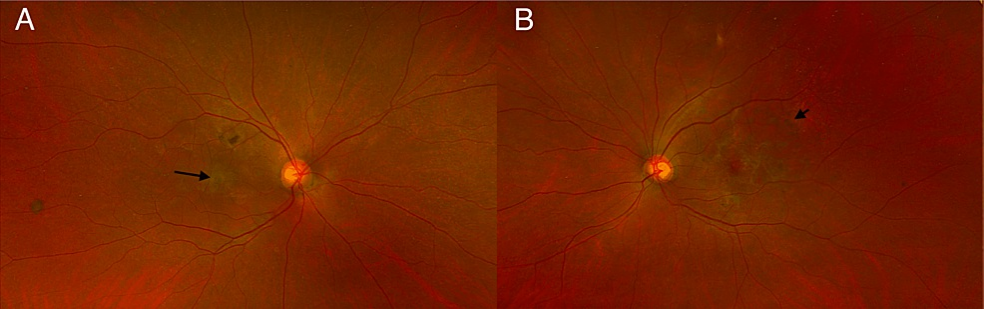
Fundus photographs A: Pigmentary changes in the fovea (black arrow), B: Pigmentary changes on the macula temporally

The patient presented to the ophthalmology consultation for blurry vision six months after the initiation of neratinib. Ophthalmological examination revealed a best corrected visual acuity (BCVA) of 20/300 (PH: 20/200) right eye and 20/25 in the left eye. Intraocular pressure of 15 mmHg in both eyes. The anterior segment exam was unremarkable with posterior chamber intra-ocular lenses. The posterior segment presented with an optic nerve that was pink and sharp with normal cup-to-disc ratios in both eyes. The macula revealed pigmentary changes; with some areas of hyperpigmentation in the macula in the right eye. The hyperpigmentation in the left eye presented a more diffuse appearance with a lacy pattern (Figure 1 A). Of note, the scarring and pigmentary clumping extended beyond the arcades in a reticular and lacy fashion in the left eye (Figure 1 B).

**Figure 2:**
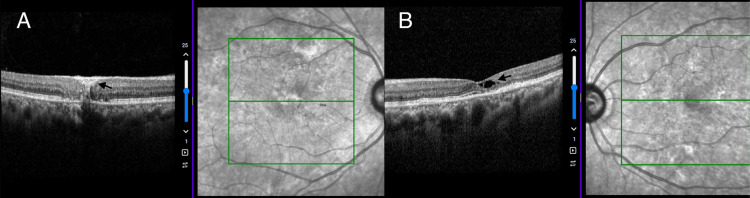
Optical coherence tomography A: Full-thickness scarring (black arrow), B: Internal limiting membrane (ILM) draping and cavitations (black arrow)

The left eye presented with more profound changes in comparison to the fellow eye. Optical coherence tomography revealed full-thickness scarring mostly of the outer retina in the right eye while presenting internal limiting membrane (ILM) draping and cavitations (Figure 2).

**Figure 3:**
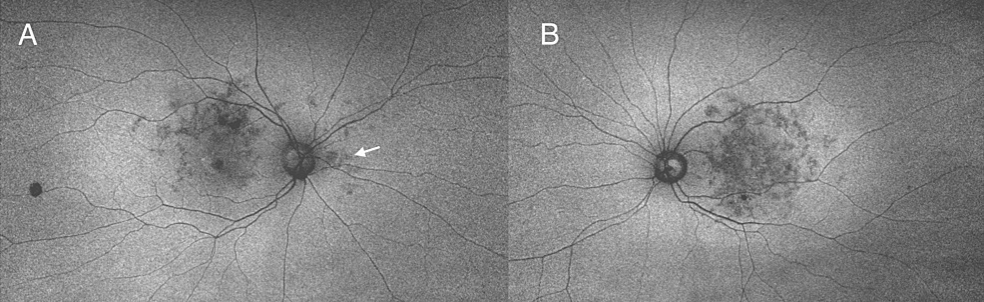
Fundus autofluorescence A: Hypo-autofluorescence within the macular and nasal to the optic nerve (white arrow), B: Hypo-autofluorescence spanning the full length of the macula

Fundus autofluorescence revealed hypoautofluorescense in areas of pigmentary clumping that extended beyond the arcades. The right eye presented similar features from the nasal to the optic disc as well (Figure 3).

**Figure 4:**
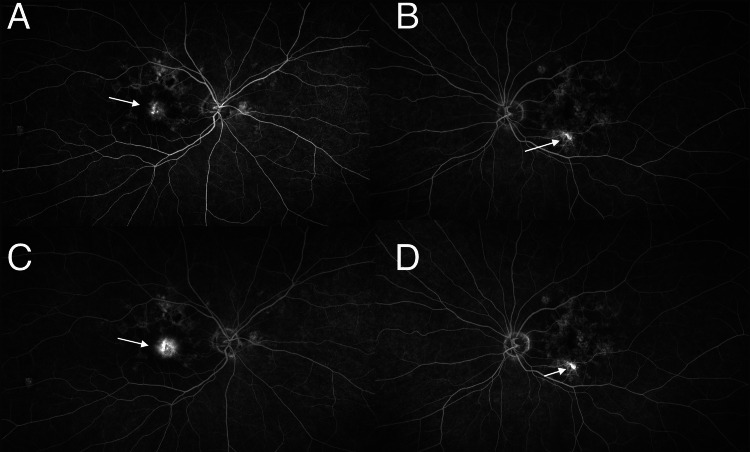
Fluorescein angiography A, B: Early hyperfluorescence from a lesion in the fovea (white arrow); C, D: Leakage in latter frames from lesions and cysts in the macula (white arrow)

Fluorescein angiography revealed early staining and late leakage beyond the area of foveal scarring in the right eye (Figure 4). There was leakage also present from a smaller lesion inferiorly on the macula in the left eye (Figure 4 D). 

## Discussion

In this case, maculopathy was noted at the sixth-month mark after the initiation of neratinib. Our patient underwent treatment for a grade 3 invasive ductal carcinoma and had previously received six cycles of docetaxel and trastuzumab. The patient developed a disease process similar to the features of MacTel type 2 after six months of neratinib therapy. There are multiple hypotheses as to how this could have occurred. One possibility is that neratinib itself could have caused the macular toxicity, given the findings and timeline of the presentation. While maculopathy is not a common side effect of TKIs, other therapeutic agents within the TKI family that have a similar mechanism of action to neratinib have been found to cause retinal toxicity [5]. Specifically, imatinib has been reported to cause optic neuritis, optic nerve edema, and cystoid macular edema. The mechanism of action leading to maculopathy with the use of imatinib or neratinib could be posited through tyrosine kinase receptor phosphorylation and accumulation in Müller cells during retinal neuronal preservation [4]. Additionally, vision loss improved to near baseline with early cessation of imatinib therapy [6,7]. Thus, previous reports of maculopathy due to imatinib use and recovery of variable vision loss with cessation of therapy promote consideration of neratinib-induced maculopathy in our patient. 

A second possibility is that the underlying macular edema could have already been present, as the patient had undergone six cycles of docetaxel chemotherapy in the past and the macular changes were subtle enough to not produce recognizable changes in the visual acuity with late outer retinal scarring. The fluorescein angiography (FA) findings do not support docetaxel toxicity given the angiographic leakage in the right eye. Docetaxel typically presents with cystoid macular edema that typically does not leak on FA [2,8]. Optical coherence tomography (OCT) with taxane use presents with cystoid space fluid accumulation, specifically within the inner plexiform layer. The mechanism of this fluid accumulation is unclear, but one pathophysiological basis could be that the agent damages Müller cells, which are responsible for maintaining the osmotic and structural homeostasis of the retina, resulting in consequential fluid accumulation and the onset of edema. In our patient, the cavitary appearance and ILM draping were more similar to the features of MacTel type II [2,4]. However, the initiation of neratinib may have served to exacerbate the ongoing disease process initially incited by docetaxel. 

Another less likely possibility is that trastuzumab was the cause of the maculopathy. However, previous reports state that after three months of initiation of trastuzumab, the maculopathy consisted of hemorrhages and hard macular exudates bilaterally which were not apparent in our patient. Additionally, the early phase FA showed a large foveal avascular zone, while late phase FA displayed leakage from the capillaries surrounding the fovea [9]. Spectral-domain OCT displayed bilateral cystoid macular edema with serous retinal detachments in patients receiving trastuzumab therapy. This finding again differs from the presentation of our patient, who instead presented with outer retinal changes, deposits, and no subretinal fluid [9].

Lastly, this case could be an atypical presentation of MacTel type II. Typically, MacTel type II presents with foveal or perifoveal ectatic vessels with the possible presence of venules diving at a right angle into deeper retinal layers. The cavitations are thought to be due to missing cells creating a void, rather than fluid actively creating cysts. Often, only the internal limiting membrane is left in place over these areas, lending to the term 'ILM drape.' While these characteristic MacTell type II features overlapped with the findings in our patient, there were also a few alterations not commonly seen with MacTell type II. Changes beyond the vascular arcades in a symmetric pattern and pigmentary changes in the nasal retina were identified in our patient, findings that are not evidenced to be associated with Mac Tel type II pathology [10].

In our opinion, we believe there to be enough evidence to support neratinib being the cause of macular toxicity in our patient considering previous treatment. Tyrosine kinase receptors have a vital role in the growth and cell recognition. Müller glial cells have unusually active protein tyrosine phosphorylation in the neural retina. The Müller glia function to balance the neuronal firing within the cell environment; the normally dormant Müller cell can upregulate its proliferation in several retinal pathologies [4]. Immunoelectron microscopy of the outer avian retina with phosphotyrosine antibodies revealed that phosphorylated tyrosine kinases accumulate mainly in Müller glia and that they are located in the Müller glial plasma membrane at sites of contact with adjacent Müller glial processes and retinal pigment epithelial photoreceptors. Thus, a proposed mechanism for neratinib maculopathy, and perhaps even toxicity, is via retinal pigment epithelial cells of the eye in which Rubicon-dependent phagocytosis and LC3-associated phagocytosis (LAP) ERBB1 signaling take place in the junction of Müller cells and the retinal pigmented epithelial cell. This signal transduction increases mammalian target of rapamycin (mTOR) phosphorylation, leading to suppression of the classic pathway and prevention of ERBB1 activation causing retinal pigment epithelium disruption [11,12].

We believe that cocktails of anti-tumoral medications such as neratinib can exacerbate macular toxicity, especially when used in tandem with other medications known to cause maculopathy such as those in the taxane class [2,9,13]. A synergistic maculopathic effect can be hypothesized given the down-regulation of neratinib on cytochrome CYP3A. Docetaxel depends mostly on CYP3A4 activity for clearance. Individual CYP3A4 activity can play a role in toxicity in those patients with an impaired CYP3A4 system, or those prone to cytochrome inhibition by certain medication use. Hepatic CYP3A4 activity can be measured by the [14C-N-methyl]-erythromycin breath test (ERMBT), which in retrospect would have been beneficial to obtain from our patient [14,15]. 

## Conclusions

Neratinib is part of the chemotherapeutic drug class known as TKIs. Their mainstay of use is for breast cancer, prostate cancer, and non-small cell lung cancer. Patients started on neratinib therapy should be counseled for rare, but possible drug-associated toxic maculopathy. This should be considered in all patients complaining of visual disturbance on neratinib therapy. An increased risk of maculopathy can be associated with concomitant taxane use. A baseline retinal examination including fundus photos and OCT of the macula should be obtained in patients undergoing treatment by taxanes, neratinib, and especially in patients undergoing combination therapy with the previous agents. The ERMBT test could be obtained in patients who are known for slow metabolizes, hepatic damage, or those undergoing treatment with medications that affect similar cytochromes. 
